# Cancer Stem Cell-Inducing Media Activates Senescence Reprogramming in Fibroblasts

**DOI:** 10.3390/cancers12071745

**Published:** 2020-06-30

**Authors:** Patrick M. Perrigue, Magdalena Rakoczy, Kamila P. Pawlicka, Agnieszka Belter, Małgorzata Giel-Pietraszuk, Mirosława Naskręt-Barciszewska, Jan Barciszewski, Marek Figlerowicz

**Affiliations:** 1 Institute of Bioorganic Chemistry of the Polish Academy of Sciences, Zygmunta Noskowskiego 12/14, 61-704 Poznań, Poland; zielinska_magda@o2.pl (M.R.); pawlicka_kamila@wp.pl (K.P.P.); abelter@ibch.poznan.pl (A.B.); giel@ibch.poznan.pl (M.G.-P.); miroslawa.barciszewska@ibch.poznan.pl (M.N.-B.); jbarcisz@ibch.poznan.pl (J.B.); marekf@ibch.poznan.pl (M.F.); 2 NanoBioMed Center, Adam Mickiewicz University, Wszechnicy Piastowskiej 3, 61-614 Poznań, Poland

**Keywords:** cellular senescence, cancer stem cells, epigenetics, chromatin, reprogramming

## Abstract

Cellular senescence is a tumor-suppressive mechanism blocking cell proliferation in response to stress. However, recent evidence suggests that senescent tumor cells can re-enter the cell cycle to become cancer stem cells, leading to relapse after cancer chemotherapy treatment. Understanding how the senescence reprogramming process is a precursor to cancer stem cell formation is of great medical importance. To study the interplay between senescence, stemness, and cancer, we applied a stem cell medium (SCM) to human embryonic fibroblasts (MRC5 and WI-38) and cancer cell lines (A549 and 293T). MRC5 and WI-38 cells treated with SCM showed symptoms of oxidative stress and became senescent. Transcriptome analysis over a time course of SCM-induced senescence, revealed a developmental process overlapping with the upregulation of genes for growth arrest and the senescence-associated secretory phenotype (SASP). We demonstrate that histone demethylases jumonji domain-containing protein D3 (Jmjd3) and ubiquitously transcribed tetratricopeptide repeat, X chromosome (Utx), which operate by remodeling chromatin structure, are implicated in the senescence reprogramming process to block stem cell formation in fibroblasts. In contrast, A549 and 293T cells cultured in SCM were converted to cancer stem cells that displayed the phenotype of senescence uncoupled from growth arrest. The direct overexpression of DNA methyltransferases (Dnmt1 and Dnmt3A), ten-eleven translocation methylcytosine dioxygenases (Tet1 and Tet3), Jmjd3, and Utx proteins could activate senescence-associated beta-galactosidase (SA-β-gal) activity in 293T cells, suggesting that epigenetic alteration and chromatin remodeling factors trigger the senescence response. Overall, our study suggests that chromatin machinery controlling senescence reprogramming is significant in cancer stem cell formation.

## 1. Introduction

Cellular senescence is a permanent cell proliferation arrest that is triggered by stress, which includes telomere shortening, oncogene expression, and DNA damage [1]. To identify cellular senescence, several biomarkers have been developed to distinguish it from other cellular events. Senescent cells have increased beta-galactosidase activity termed SA-β-gal [2]. To apprehend proliferation, the activation of many tumor suppressor proteins are engaged in senescent cells, including p16INK4A, p15INK4B, p21CIP1, and p53 [3]. A large body of evidence demonstrates cellular senescence as a barrier to tumorigenesis [4]. Interestingly, cancer cells exposed to radiation or chemotherapy can still enter a state of senescence-growth arrest [5]. More recently, it has been demonstrated that cancer stem cells arise from senescent tumor cells that re-enter the cell cycle [6]. The cells formed from this process retain senescence-like traits, along with their tumor-initiating potential. Cellular senescence is known to block normal functioning cells from adopting plasticity between cell fates. For example, it prevents the generation of induced pluripotent stem cells (iPSCs) from adult fibroblasts [7]. However, it remains largely unknown how the senescence reprogramming process contributes to cancer stem cell formation.

Several studies indicate that chromatin remodeling is critical for senescence reprogramming. Condensed chromatin structures, known as senescence-associated heterochromatin foci (SAHF), are a defining feature of the senescent cell nucleus [8]. SAHF are thought to be specialized domains of facultative heterochromatin that contribute to the silencing of proliferation-promoting genes. At the same time, local gene up-regulation of key senescence genes exists within the chromatin landscape, including those involved in the senescence-associated secretory phenotype (SASP) [9]. The robust changes in gene expression during senescence are attributed to the downregulation of the nuclear lamina-associated protein, lamin B1, and the loss of the epigenetic repressive mark tri-methylation of Lys 27 on histone H3 (H3K27me3) in chromatin [10]. We hypothesize that such epigenetic alteration events and chromatin remodeling found in senescent cells are implicated in the formation of cancer stem cells.

Jumonji domain-containing protein D3 (Jmjd3) and X chromosome (Utx) are histone demethylases specific for removing methyl groups from H3K27me3 [11]. They are both negative regulators of the somatic cell reprogramming process. For example, the transcription factors, Oct4, Sox2, Klf4, and Myc, can reprogram somatic cells to iPSCs, however, without the presence of Jmjd3 or Utx, cannot facilitate iPSC formation [12,13]. Jmjd3 demethylase activity has already been identified as a means of regulating oncogene-induced senescence [14]. Meanwhile, Utx regulates lifespan in *C. elegans* [15] and enables tumor suppression via cell fate control [16]. This evidence suggests the role of Jmjd3 and Utx in activating senescence reprogramming and cell fate mechanisms in normal cells.

Previously, a media containing, B27 supplement, epidermal growth factor (EGF), and fibroblast growth factor (FGF), denoted here as stem cell media (SCM), has been used to propagate several stem cell types, including neural stem cells [17]. SCM is known to be cancer-stem cell inducing, as it activates stem cell markers and properties such as self-renewal and clonogenicity in cancer cells [18]. The SCM culturing conditions over time select for stem-like traits that more closely mimic the phenotype of primary tumors [19]. This has also been referred to as floating culture conditions, as it changes the growth and characteristics of cells from adherent to anchorage-independent spheres.

Here, we cultured human embryonic fibroblasts in SCM and found that it activates a developmental process, along with characteristic features of cellular senescence. The mechanism involves histone demethylases Jmjd3 and Utx proteins. By assessing senescence-associated biomarkers in cancer cell lines cultured in SCM, we found that similar senescence reprogramming is inherently part of the cancer stem cell phenotype. 

## 2. Results

### 2.1. SCM Triggers Spontaneous Oxidation of DNA and Nuclear Blebbing

**Figure 1:**
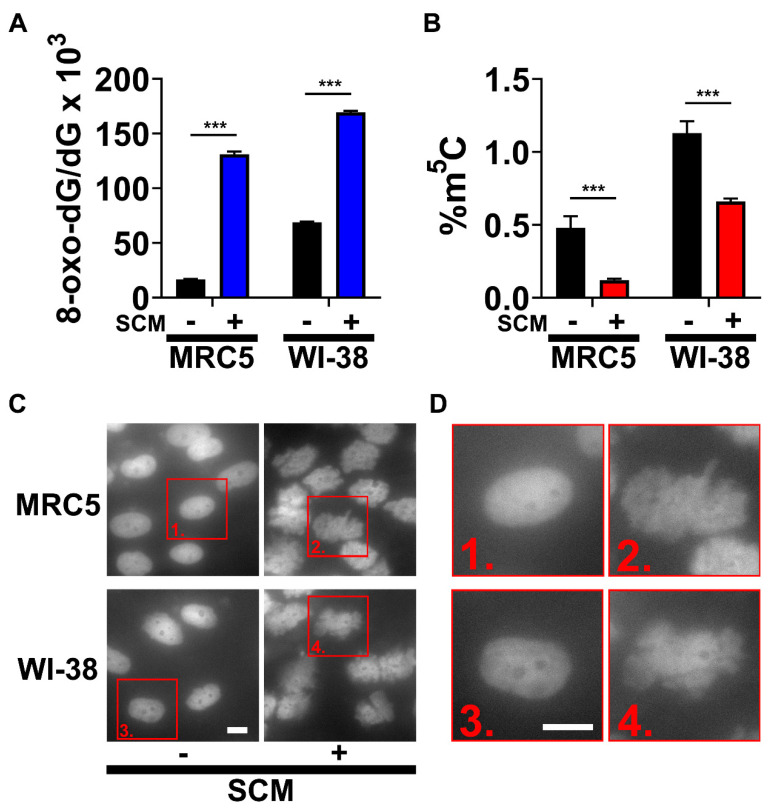
DNA oxidation and nuclear blebbing in WI-38 and MRC5 cells. (**A**) Quantification of 8-oxo-dG in DNA at 24 h using electrochemical detection. (**B**) Quantification of m^5^C in DNA at 24 h using a thin-layer chromatography method. (**A**,**B**) Student’s *t*-test; bars represent standard deviation and asterisks indicate statistical significance (***, *p* ≤ 0.001; *n* = 4). (**C**) Representative fluorescence microscopy images of stained nuclei at 36 h. (**D**) Nuclei labeled 1–4 from **C** are enlarged (scale bars, 10 µm).

We tested the effects of SCM on human embryonic fibroblasts (MRC5 and WI-38) and found that it induced robust cellular senescence. An early feature of SCM-induced senescence is oxidative stress (damage) caused by ROS and nuclear blebbing. To detect DNA oxidation, we isolated and analyzed DNA for changes in modifications. Notably, 8-oxo-deoxyguanosine (8-oxo-dG) is a product of the hydroxyl radical (•OH) reaction with guanosine (G) at position C-8, and a specific marker of ROS-driven DNA-damage [20]. The levels of 8-oxo-dG significantly increased in MRC5 and WI-38 after 24 h in SCM (Figure 1A). Since oxidative damage can also occur with all DNA constituents, we concomitantly analyzed 5-methylcytosine (m^5^C) in the same DNA samples. Moreover, m^5^C is an epigenetic mark in DNA that modulates gene expression [21]. Contrary to 8-oxo-dG, the level of m^5^C significantly decreased in MRC5 and WI-38 after 24 h in SCM (Figure 1B). This data suggests that SCM-treated fibroblasts spontaneously generate ROS that reacts with global DNA, resulting in the oxidation of G and m^5^C demethylation. 

Nuclear blebbing is a mechanism of nuclear reorganization in senescent cells [22]. To detect the occurrence of this process in SCM-induced senescence, we stained nuclei using a fluorescent dye specific for DNA (Figure 1C,D). Misshapen nuclei were prevalent in SCM-treated fibroblasts, and the duration of the nuclear blebbing was variable between 36–48 h. These results suggest a massive ‘chromatin relaxation’ (opening), which can be seen as protrusions from the nuclear surface.

### 2.2. The Emergence of Senescence-Associated Biomarkers in SCM-Treated Fibroblasts

**Figure 2:**
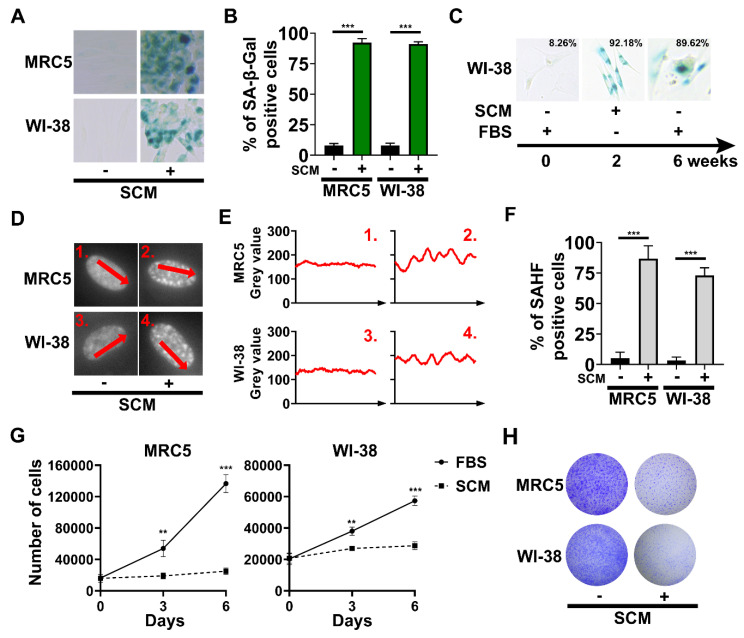
Senescence-associated biomarkers in MRC5 and WI-38 cells treated with stem cell medium (SCM). (**A**) Representative images for senescence-associated beta-galactosidase (SA-β-gal) staining at 48 h. (**B**) Quantification of SA-β-gal shown in **A**. (**C**) WI-38 cells grown in SCM for two weeks, followed by 10% FBS media for four weeks. Representative images for SA-β-gal staining at each timepoint condition. Numbers in the upper-right corner of each picture represent the percent of SA-β-gal positive cells (**D**) Representative images of stained nuclei at 72 h. (**E**) Linescan analysis (ImageJ software) of fluorescence intensity profiles along the arrows 1–4 indicated in **D**. (**F**) Quantification of cells with senescence-associated heterochromatin foci (SAHF)**.** (**G**) Cell proliferation curve of MRC5 and WI-38 cells grown in FBS and SCM. A total of 15,000 cells were plated overnight in a 12-well cell culture plate. Counting began on the following day after the cells became attached to the bottom of the wells. Cell nuclei were stained with Hoechst 33342 and counted based on fluorescence microscopy images taken at each time point. (**H**) Images showing crystal violet staining of cells grown in SCM for three days, followed by 10% FBS media for 20 days. (**B**, **F**, and **G**) Student’s *t*-test; bars represent standard deviation and asterisks indicate statistical significance (**, *p* ≤ 0.01; ***, *p* ≤ 0.001; *n* = 3).

We characterized senescence-associated biomarkers at later time points. MRC5 and WI-38 cells, after 48 h SCM-treatment, had significantly increased SA-β-gal activity (Figure 2A,B). Long-term culture of WI-38 cells in SCM for 2 weeks followed by culturing in FBS media for 4 weeks (6 weeks total) showed that SA-β-gal activity was persistent (Figure 2C). Next, we fluorescently stained MRC5 and WI-38 cell nuclei at 72 h, to detect the presence of SAHF. SCM-treated fibroblasts exhibited a greater overall fluorescence intensity and variably sized puncta in their nuclei (Figure 2D,E). Overall, the number of cells with SAHF significantly increased compared to the controls (Figure 2F). SA-β-gal activity and SAHF prompted us to check whether SCM-treated fibroblasts were permanently growth-arrested. Proliferation assays demonstrated that SCM significantly inhibited proliferation by 3 days, with continued growth arrest observed out to 6 days (Figure 2G). We also assessed proliferation by releasing SCM-treated fibroblasts back into media which contained FBS. After subsequent passaging and 20 days in FBS media, SCM-treated fibroblasts never reached a confluency of the dish (Figure 2H). Importantly, we show that control cells still proliferated, even after a 72 h period of FBS starvation. The inability to proliferate in response to growth media, corroborated by SA-β-gal activity and SAHF, strongly suggests that SCM-induced senescence is a permanent growth-arrested state.

### 2.3. Chromatin Remodeling and Robust Changes in Gene Expression during SCM-Induced Senescence

**Figure 3:**
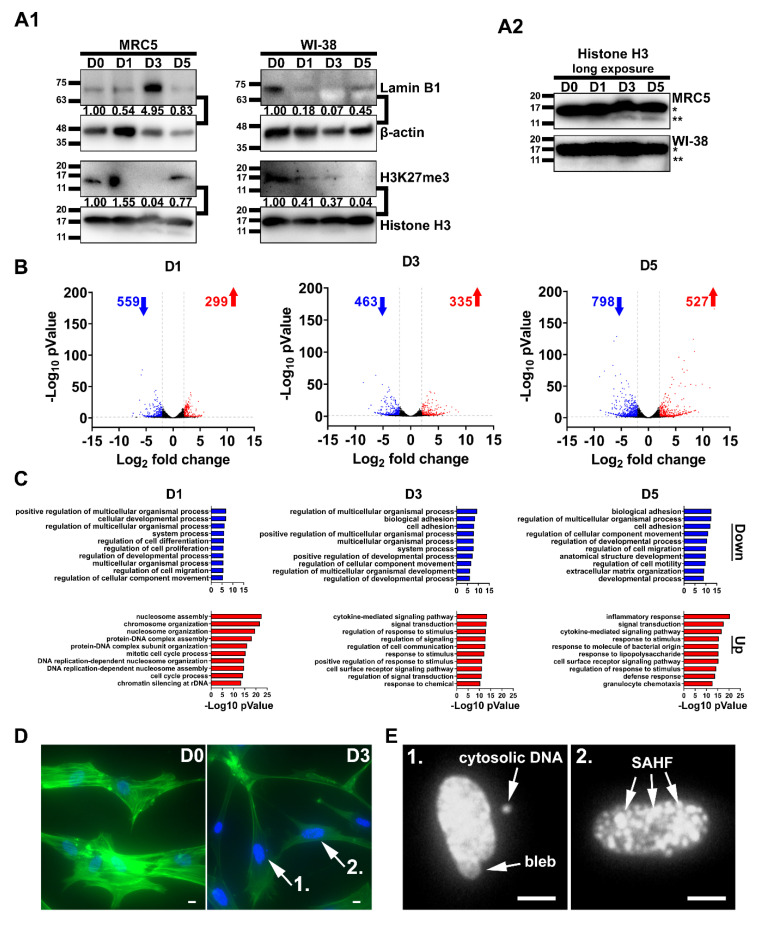
Dynamic chromatin remodeling and gene expression in SCM-induced senescence. (**A1**) Western blot showing the variation in lamin B1 and H3K27me3 levels in MRC5 and WI-38 cells over a five-day time course. The number below each band is the densitometry value (intensity ratio) compared to β-actin or histone H3 and relative to the control condition. (**A2**) Long exposure of Histone H3 loading control (* full-length Histone H3, ** cleaved). (**B**) Time course RNA-sequencing of MRC5 cells undergoing SCM-induced senescence. Volcano plots show differential gene expression between control (D0) and SCM-treated samples (D1, D3, and D5). The sequencing of RNA was performed in triplicate for each time point. The number of genes up- (red) and down-regulated (blue) are labeled on each plot. (**C**) GO enrichment analysis of transcriptome data. The up- (red) and down-regulated (blue) genes in the plots in **B** were all used in the analysis. The graphs show the top-10 biological processes affected by SCM conditions at each time point. (**D**) MRC5 cells stained for the nucleus (blue) and F-actin (green) at D0 and D3 (Scale Bar, 10 µm). (**E**) Nuclei labeled 1 and 2 from **D** are enlarged and presented in greyscale to show nuclear blebbing, cytosolic DNA, and SAHF (Scale Bar, 10 µm).

We performed a time course experiment to monitor the distinctive global changes to chromatin during SCM-induced senescence. Protein lysates were taken from MRC5 and WI-38 cells cultured in SCM for 0, 1, 3, and 5 days (denoted as; D0, D1, D3, and D5), and subsequently immunoblotted for lamin B1 and H3K27me3 (Figure 3A1). We observed cell-type-specific patterns for these markers. The protein levels of lamin B1 showed dynamic fluctuations in MRC5 cells, versus a complete loss in WI-38 cells. We observed marked decreases in H3K27me3, in both MRC5 and WI-38 cells by D3. However, while remaining depleted in WI-38 cells, global H3K27me3 levels were reestablished at D5 in MRC5 cells. Interestingly, the histone H3 loading control at 17kDa (*) had a faint extra lower molecular weight band (**) that did not appear in the control, D0 sample (Figure 3A2). We speculate that the extra lower molecular weight band corresponds to the N-terminus of H3 variants reported to be cleaved during senescence [23]. Overall, our results reflect the chromatin dynamics consistent with senescence reprogramming in MRC5 and WI-38 cell lines.

Next, we performed RNA-sequencing to take a snapshot of the transcriptional changes in MRC5 cells undergoing SCM-induced senescence. Using the control (D0) as a reference, we identified differentially expressed genes at each subsequent time point (D1, D3, D5). The number of upregulated (D1 = 299, D3 = 335, D5 = 527) and downregulated (D1 = 559, D3 = 463, D5 = 798) genes were determined on the basis of a ≤ log2-fold change value of 2, and a *p*-value ≤ 0.05 (Figure 3B and Table S1). In addition to this, we also performed a time course analysis that combines the data sets for every time point relative to D0 (Table S2). To identify biological processes occurring during SCM-induced senescence, we used the significantly up- and down-regulated genes at each time point for GO enrichment analysis (Figure 3C and Table S3). Consistent with our observations of morphological changes to the nuclei shown in Figure 1C, the top biological pathways defined at D1 for upregulated genes included nucleosome assembly (GO:0006334; *p*-value = 2.83 × 10^−23^) and chromosome organization GO:0051276; *p*-value = 1.79 × 10^−22^). Claspin was the second-highest upregulated gene in the dataset at D1 (log2-fold change = + 5.31; *p*-value = 0.0005). Interestingly, claspin represents a regulation of the mitotic G2 DNA damage checkpoint, previously linked to senescence growth arrest [24]. The expression of claspin after D1 returns to baseline levels, suggesting that it is part of the early response to SCM. The cell cycle regulators p15INK4B (log2-fold change = + 3.93, *p*-value = 7.75 × 10^−10^) and p21CIP1 (log2-fold change = + 2.65, *p*-value = 9.00 × 10^−5^) were significantly increased in the time course analysis, in line with our observations of growth-arrest. Notably, hairy and enhancer of split-1 (HES1), an important transcription factor for stemness and differentiation [25], was upregulated (log2-fold change = + 5.13, *p*-value = 5.14 × 10^−14^).

The top GO pathways for upregulated genes at D3 and D5 were “cytokine-mediated signaling pathway” (GO: 0019221; *p*-value = 3.61 × 10^−14^) and “inflammatory response” (GO:0006954; *p*-value = 4.56 × 10^−21^). These pathways contain the inflammatory cytokines, IL-1A, IL-1B, IL-6, and IL-8, that overlap with the SASP. Notably, these genes steadily increase in expression starting at D3 and spike at D5, indicating that the SASP is gradually activated in SCM-induced senescence. GO pathways for downregulated genes were “regulation of multicellular organismal process” (GO:0051239; *p*-value = 3.71 × 10^−10^) at D3, and ‘biological adhesion” (GO:0022610; *p*-value = 3.27 × 10^−13^) at D5. Moreover, synaptopodin-2 (SYNPO2), a muscle-related gene, was downregulated (log2-fold change = −7.30, *p*-value = 3.94 × 10^−102^). SYNPO2 was previously shown to interact with stress fibers of the cytoskeleton which are involved in focal adhesion [26]. We stained for F-actin and observed fewer stress fibers at D3 compared to D0 (Figure 3D). We also detected, in these cells, cytosolic DNA, along with SAHF, which corroborates a nuclear blebbing process (Figure 3E). Altogether, the changes in gene pathways and the cytoskeleton indicate a developmental process concomitant with cellular senescence in MRC5 cells.

### 2.4. Inhibition of H3K27me3 Demethylases Prevents SCM-induced Senescence in Fibroblasts

**Figure 4:**
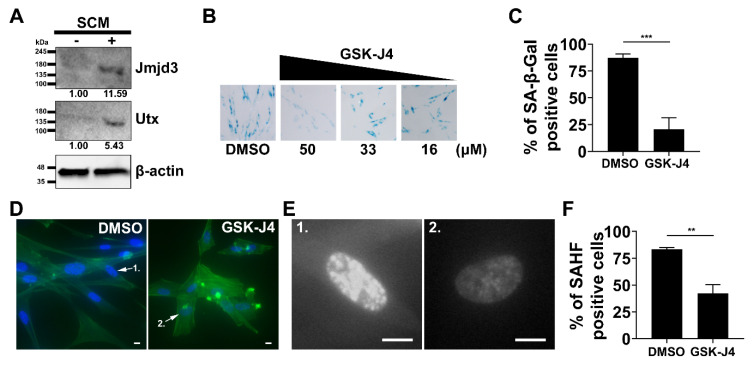
Chemical inhibitor GSK-J4 blocks the effects of SCM. (**A**) Western blot showing Jmjd3 and Utx levels in MRC5 cell lysates after 48 h in SCM. The number below each band is the densitometry value (intensity ratio) compared to β-actin and relative to the control condition. (**B**) MRC5 cells simultaneously treated with SCM and GSK-J4 at different concentrations. Representative images for SA-β-gal staining at 48 h. DMSO was the vehicle control. (**C**) Quantification of SA-β-gal shown in **B** for 50 µM of GSK-J4 compared to DMSO. (**D**) Representative images MRC5 cells stained for the nucleus (blue) and F-actin (green). Cells were treated simultaneously with SCM and GSK-J4 (50 µM) for 72 h. (Scale Bar, 10 µm). (**E**) Cell nuclei 1 and 2 from **D** are enlarged and presented in greyscale (Scale Bar, 10 µm). (**F**) Quantification of cells with SAHF at 72 h. (**C**,**F**) Student’s *t*-test; bars represent standard deviation and asterisks indicate statistical significance (**, *p* ≤ 0.01; ***, *p* ≤ 0.001; *n* = 3).

We investigated the role of H3K27 histone demethylases Jmjd3 and Utx in SCM-induced-senescence. Immunoblotting shows the presence of Jmjd3 and Utx proteins in MRC5 cells after 48 hr treatment in SCM (Figure 4A). We tested whether these enzymes were necessary for SCM-induced senescence by applying GSK-J4, a chemical inhibitor for H3K27 demethylases [27]. MRC5 cells were simultaneously treated with SCM and a range of GSK-J4 concentrations (16, 33, and 50 μM). A significant inhibitory effect on SA-β-gal activity was observed at a concentration of 50 μM (Figure 4B,C). GSK-J4 also prevented SAHF and a loss of cytoskeleton stress fibers (Figure 4D–F). This data shows that GSK-J4 could block some of the effects of SCM on MRC5 cells.

**Figure 5:**
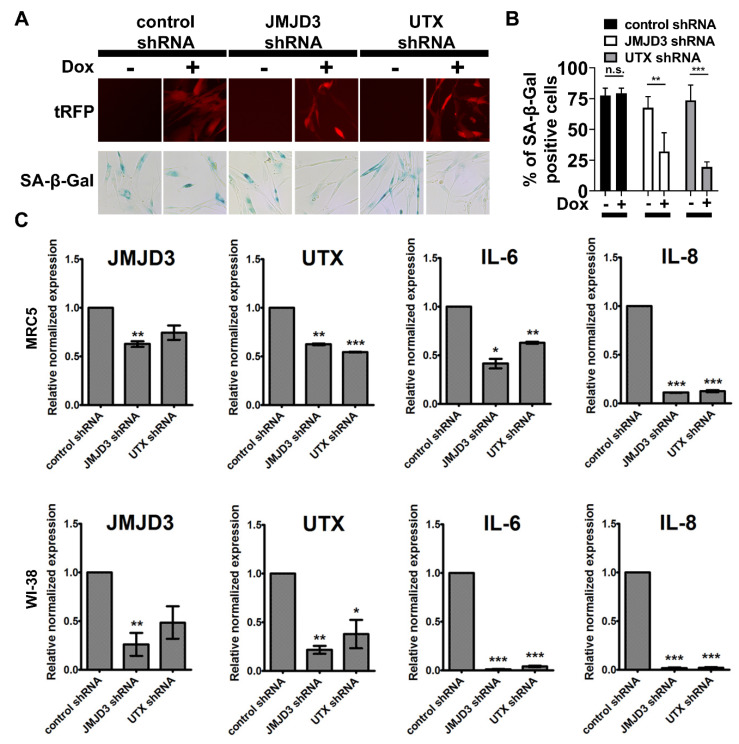
shRNA knockdown of H3K27 demethylases reduces senescence-associated biomarkers. (**A**) Representative fluorescence microscopy images of MRC5 cell lines grown in SCM and expressing shRNAs targeting Jmjd3 and Utx. The addition of doxycycline (Dox) at 2 µg/mL activated each shRNA, as indicated by turbo red fluorescent protein (tRFP) reporter expression. Representative images show SA-β-gal staining at 48 h. (**B**) Quantification for SA-β-gal staining shown in A. (**C**) RT-qPCR analysis for the expression levels of H3K27 demethylases and SASP genes in control, Jmjd3, and Utx shRNA cell lines at 72 h. (**B**,**C**) Student’s *t*-test; bars represent standard deviation and asterisks indicate statistical significance (n.s., not significant; *, *p* ≤ 0.05; **, *p* ≤ 0.01; ***, *p* ≤ 0.001; *n* = 3).

Next, we performed individual RNAi knockdowns of Jmjd3 and Utx, to identify their roles in SCM-induced senescence. Doxycycline-inducible shRNA constructs were packaged into lentivirus and fibroblasts were subsequently infected and selected for based on puromycin resistance. In comparison to shRNA and doxycycline controls, Jmjd3 or Utx gene knockdown significantly reduced the emergence of SA-β-gal activity in MRC5 cells. (Figure 5A,B). Furthermore, RT-qPCR was performed to analyze the global levels of Jmjd3, Utx, and their relationship to the expression of SASP in MRC5 and WI-38 cells (Figure 5C). We found that SASP factors, IL-6 and IL-8, were significantly reduced with a loss of function of Jmjd3 and Utx. These data suggest that methyl-modifications and chromatin regulation related to the function of Jmjd3 and Utx play an important role in the activation of SCM-induced senescence.

### 2.5. Chromatin Machinery for Senescence Reprogramming is Activated in Cancer Stem Cell Formation

**Figure 6:**
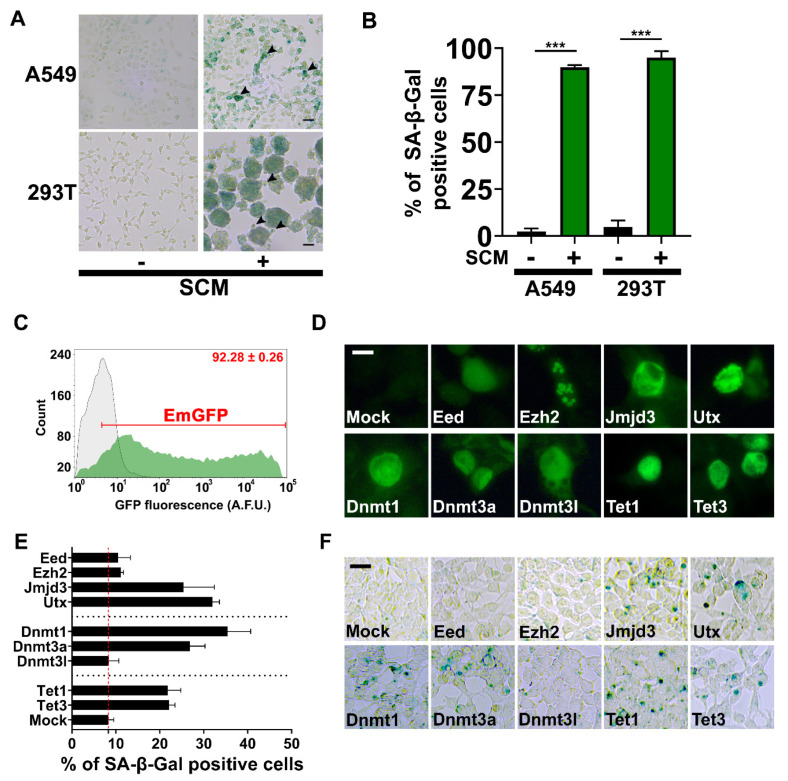
Cancer stem cells and 293T cells overexpressing H3K27 demethylases display SA-β-gal+. (**A**) Representative images of A549 and 293T cancer stem cells stained for SA-β-Gal after seven days in SCM. Arrowheads point to anchorage-independent cells with SA-β-Gal+ (Scale Bars, A549 = 50 µm, 293T = 100 µm). (**B**) Quantification for SA-β-gal staining. Student’s *t*-test; bars represent standard deviation and asterisks indicate statistical significance (***, *p* ≤ 0.001; *n* = 3). (**C**) The mean transfection efficiency percentage of 293T cells was determined using a DNA construct encoding emerald green fluorescent protein (EmGFP). Cells were analyzed at 48 hr post-transfection by flow cytometry in triplicate. Flow cytometry histograms for transfected cells (green) and mock control (grey) are overlaid on the plot. The mean percentage of EmGFP+ cells was calculated using the gate limit setting of the mean fluorescence intensity of the mock control (number in the upper right corner in red is the percentage of EmGFP positive cells ± standard deviation). (**D**) Fluorescence microscopy images of transfected cells expressing the indicated proteins. Expression was detected by immunofluorescence staining (HA, FLAG, and c-myc fusion tags), followed by fluorescence microscopy (Scale Bar, 10 µm). (**E**) The graph shows the percentage of SA-β-gal+ at 48 hr post-transfection; mean ± SD were calculated from three different fields of view from images containing ≥300 cells. A mock transfection (no DNA) was the control. The red dashed line is equal to the mean value of the mock control for reference. (**F**) Representative bright-field images of SA-β-gal stained cells that were used for quantification in E (Scale Bar, 25 µm).

Using a method to isolate cancer stem cells, we cultured non-small-cell lung cancer (A549) and HEK293 cells transformed with large T antigen (293T) cells in SCM. Anchorage-independent cells and sphere formation indicated the presence of cancer stem cells at seven days, as previously described [28]. Both 293T and A549 cancer stem cells had robust SA-β-gal activity compared to low levels of staining in the controls (Figure 6A,B). Next, we implemented a screening strategy to identify chromatin machinery proteins sufficient to induce SA-β-gal activity on their own, in the absence of canonical and cellular growth arrest pathways (Figure 6C–E). DNA-constructs encoding DNA methyltransferases [29], ten-eleven translocation dioxygenases [30], polycomb-group proteins [31], and H3K27 demethylases were transfected into 293T cells. We found that DNA methyltransferases, Dnmt1 and Dnmt3a, which can produce m^5^C in DNA, increased the percentage of SA-β-gal+ cells compared to DNA methyltransferase 3-like (Dnmt3l), which does not possess any inherent enzymatic activity. Ten–eleven translocation dioxygenases, Tet1 and Tet3, which catalyze the demethylation of m^5^C also increased the percentage of SA-β-gal+ cells. Notably, polycomb-group proteins, embryonic ectoderm development (Eed) and enhancer of zeste homolog 2 (Ezh2) weakly induce SA-β-gal activity in contrast to H3K27 demethylases, Jmjd3 and Utx. Ezh2 is the functional enzymatic component of the polycomb repressive complex 2 (PRC2), which is responsible for the tri-methylation of H3K27 during development and differentiation. Eed functions as the H3K27me3 binding subunit of PRC2, and through this interaction, enhances the enzymatic activity of Ezh2. Overall, these data indicate that H3K27me3 is a barrier to senescence reprogramming.

## 3. Discussion

SCM-induced senescence is a process to study the impact of oxidative stress on cell fate and cancer stem cell mechanisms. Our data indicate the increasing presence of 8-oxo-dG in fibroblasts after SCM-treatment. This observation strongly suggests that random ROS-induced oxidation facilitates the rapid demethylation of m^5^C and subsequent reactions coupled to histone modifications during SCM-induced senescence. The oxidative demethylation of m^5^C in the promoter regions of some genes is known to activate transcription [32]. Additionally, we observed nuclear blebbing, a mechanism for expulsing chromatin from the nucleus, into the cytoplasm for digestion [33]. Similar situations involving nuclear blebbing are observed in cancer and diseases of rapid aging, such as Hutchinson–Gilford progeria syndrome [34]. These events turn on classic features of cellular senescence in human fibroblasts, including growth arrest, SA-β-gal activity, SAHF, and SASP. 

Our data suggest that lamin B1 protein levels fluctuate over time, accounting for the detachment of DNA sequence domains from the nuclear lamina during senescence reprogramming. The loss of lamin B1 represents a senescence-associated biomarker [35]. We observed a cell-type-specific pattern for lamin B1 in MRC5 fibroblasts undergoing SCM-induced senescence. Interestingly, there are reports that cellular senescence is triggered by the overexpression or knockdown of lamin B1 [36]. These examples explain our observations in the MRC5 cell line, which exhibited increased levels of lamin B1 at D3, which eventually were downregulated by D5. In contrast, WI-38 cells showed the early downregulation of lamin B1 that remained this way throughout the 5-day time course. 

We observed both MRC5 and WI-38 cells have a global reduction of H3K27me3 by D3 of SCM treatment. A recent paper shows that the global reduction of H3K27me3 is associated with cellular senescence [37]. Interestingly, H3K27me3 marks can still be found within the structural layers of SAHF [38]. The formation of SAHF is thought to be an independent event from epigenetic remodeling. However, the erasure of H3K27me3 has profound effects that lead to cell lineage commitment [39]. In MRC5 cells, we observed H3K27me3 returns near the baseline levels at D5. Therefore, in the context of SCM-induced senescence, the removal of H3K27me3 at D3 represents a loss of cell identity, and any reestablishment/static landscape for H3K27me3 is not necessarily indicative of an improper senescence program.

The inhibition of Jmjd3 or Utx reduced the effects of SCM. The role of H3K27 demethylases is apparent in cellular senescence, because they are upregulated and recruited to p53 bound promoters and enhancer elements under stress responses [40,41]. There is a large amount of evidence showing that Jmjd3 activates robust features of cellular senescence in many cell types. Jmjd3 is necessary and sufficient for the formation of SAHF in human WI-38 fibroblasts [42]. The overexpression of Jmjd3 in U251 glioma cells activates the SASP, which contributes to tumor progression [43]. Additionally, Utx conditional knockout mice have significantly reduced SA-β-gal+ senescent cells in their skeleton during late puberty [44]. These examples, along with our data, suggest that Jmjd3 and Utx could play a role in several forms of cellular senescence. Our findings overlap with other reports, showing that the behaviors of cancer stem cells are controlled through H3K27me3 demethylase activity. For example, GSK-J4 blocks the characteristic features of breast cancer stem cells [45]. The same study also examined Jmjd3 and Utx, and found them to be necessary for expansion, self-renewal capacity, and the expression of stemness-related markers. Therefore, the demethylation of H3K27 puts cancer cells, through senescence reprogramming, on the way to cancer stem cell formation. 

Our transcriptome data analysis of MRC5 cells revealed the upregulation of p15INK4B and p21CIP1, which were previously shown to play a role in programmed senescence. Recently, it was discovered that cellular senescence can be an instructed process during development. This so-called, programmed senescence is shown to be important during embryogenesis for growth and patterning of tissues [46]. In contrast, senescent cells that accumulate in tissues during the aging process exhibit double-strand DNA breaks [47]. Interestingly, we found that the expression of claspin at D1, which indicates the engagement of checkpoint mediated cell cycle arrest in response to ssDNA, broke and stalled replication forks. Thus, more experiments are needed to examine whether this process is independent of a DNA-damage response involving p53 and p16INK4A. Nonetheless, it provides a new model for the study of senescence involvement in different cell regulation mechanisms. 

**Figure 7:**
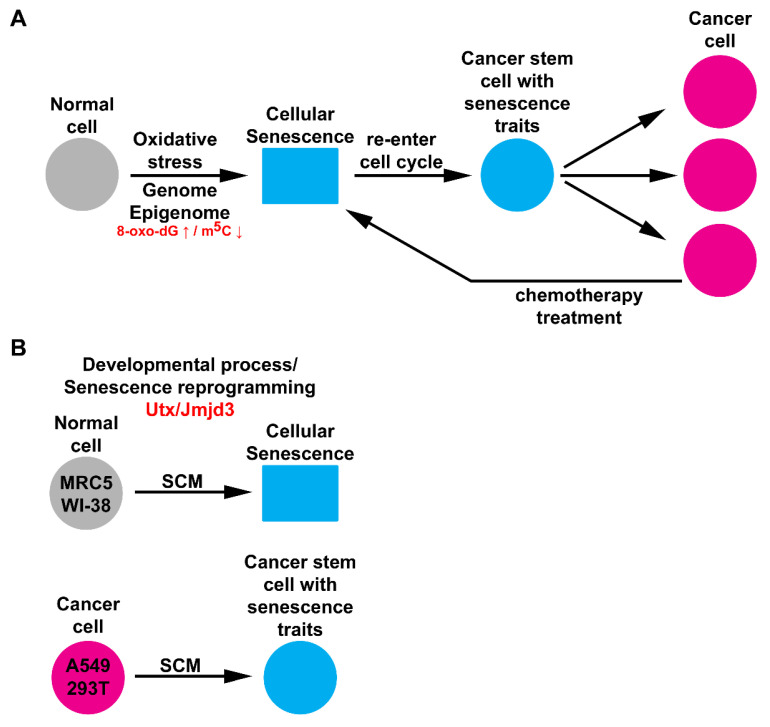
Graphical summary. (**A**) The concept of cancer development arising from the bypass of cellular senescence. Normal cells under oxidative stress accumulate genomic and epigenomic damage (8-oxo-dG ↑ and m^5^C ↓) that leads to cellular senescence. Cancer cells treated with chemotherapy can also produce senescent cells, which are essentially dormant tumor cells. It is poorly understood how senescent cells can re-enter the cell-cycle to produce cancer stem cells with senescence traits. Ultimately, cancer cells that are produced from this process contribute to tumor relapse and progression. (**B**) Data arising from this study show that SCM activates cellular senescence in MRC5 and WI-38 fibroblasts. Histone demethylases, Utx and Jmjd3, facilitate a developmental process, overlapping with senescence reprogramming. In comparison to normal cells, A549 and 293T cancer cells cultured in SCM are converted to cancer stem cells with senescence traits.

Our data suggest that SCM activates senescence reprogramming during cancer stem cell formation (Figure 7A,B). The presence of SA-β-gal activity in cancer stem cells demonstrates the uncoupling of a senescence-associated phenotype from growth arrest. Future studies will need to address how continued self-renewal impacts other senescence-associated biomarkers and its contribution to tumor progression. Chromatin machinery proteins that modify DNA and histone methylation may serve as novel targets for inhibiting this process. Widespread DNA hypomethylation and focal hypermethylation are characteristic features of both senescent and cancer cells [48]. We found that the overexpression of enzymes that act on DNA methylation could activate SA-β-gal activity in 293T cells. At the level of histones, erasers (Jmjd3 and Utx) for H3K27me3 induced SA-β-gal activity, but the reader and writer (Eed and Ezh2) did not. This is consistent with several alternative mechanisms for senescence induction via ‘chromatin relaxation’ that have been reported [49,50]. Our findings provide important clues for designing new therapies that target the epigenome (H3K27me3 mark) of cancer cells.

## 4. Materials and Methods 

### 4.1. Cell Culture

MRC5 (CCL-171), WI-38 (CCL-75), A549 (CCL-185), and 293T (CRL-3216) cell lines were obtained from American Type Culture Collection (ATCC). Cells were cultured in Eagle’s minimum essential medium (EMEM), with 1.5 g/L sodium bicarbonate, non-essential amino acids, L-glutamine, sodium pyruvate (Corning; 10-009-CV, Corning, NY, USA), 10% fetal bovine serum (EURx E5050-02, Gdansk, Poland) and antibiotic/antimycotic solution at 1X concentration (Sigma A5955, St. Louis, MO, USA). The media were filtered using a 0.22-micron PES disc syringe filter (Millipore; Cat No. S2GPU05RE, Burlington, MA, USA). All cell cultures were incubated in a humidified chamber containing 5% CO_2_ at 37 °C. Cell passaging was performed using a trypsin-EDTA solution (Sigma; T4049). The SCM components, B-27 supplement (50X) (ThermoFisher Scientific; Cat. No. 17504044, Waltham, MA, USA), fibroblast growth factor; FGF (Sigma-Aldrich; F0291, St. Louis, MO, USA), epidermal growth factor; EGF (Sigma-Aldrich; E9644) were stored as aliquots at −20 degrees, and subsequently kept at 4 °C while in use to prevent freeze and thaw cycles. The EGF and FGF were prepared for cell culture by resuspending them in the glass vials they arrived in under sterile conditions at a stock concentration of 50 µg/mL. The solvent for growth factor resuspension was a 1% BSA solution in PBS, that was filtered using a 0.22-micron PES syringe disc filter (Millipore; Cat. No. SLGP033RS). For the induction of cellular senescence, the FBS media were completely removed and replaced with only EMEM basal media with antibiotic/antimycotic. After 24 h of serum starvation, 20 µL of B-27 (50×), 1 µL of EGF and 1 µL of FGF stock solutions were added directly to every 1 mL of EMEM basal media with antibiotic/antimycotic, to achieve a final concentration of B-27 (1×), 50 ng/mL EGF and 50 ng/mL FGF. For all experiments, the SCM components were added every 24 h and a complete change of the basal media occurred every 3 days.

### 4.2. Analysis of 8-oxo-dG and m5C

Cells were first detached from the culture flask using a trypsin-EDTA solution, and centrifuged at 1200 rpm for 5 min at 4 °C. The cell pellet was washed in PBS and then immediately processed for DNA isolation using a genomic DNA extraction kit (A&A Biotechnology; Cat. No. 116–250, Gdynia, Poland), in accordance with the manufacturer’s protocol. The purity and concentration of DNA preparations were checked by measuring UV absorbance at 260 and 280 nm. The A_260_/A_280_ ratios were between 2.0–2.1.

The analysis of 8-oxo-dG was performed as previously described [51]. Briefly, DNA was treated with nuclease P1 for 3h and alkaline phosphatase for 1 h at 37 °C. The DNA hydrolysate was purified using a filter with a cut-off of 10,000 Da. Then, 8-oxo-dG and dG in hydrolysates were determined using HPLC (Agilent Technologies 1260 Infinity), with two detectors working in series: a 1260 Diode Array Detector and a Coulochem III Electrochemical Detector (ESA, Inc., Chelmsford, MA, USA). DNA hydrolysates were chromatographed isocratically using 50 mM ammonium acetate, pH 5.3/methanol (93:7). The detection of dG was performed at 254 nm. Then, 8-oxo-dG was determined by the electrochemical detector: guard cell +400mV, detector 1: +130mV (as a screening electrode), detector 2: +350mV (as a measuring electrode set to sensitivity of 50 nA/V). Moreover, 8-oxo-dG standard was purchased from Sigma-Aldrich. The input amount of guanosine was necessary to determine 8-oxo-dG contents. It was calculated from diode array detector (DAD) measurements, using Avogadro number dG = 6.02 × 10^20^ × b(mAU sample)/a(mAU standard). Furthermore, the 8-oxo-dG nucleoside amount was estimated with the formula 8-oxo-dG = 6.02 × 10^20^ × d(dG sample)/(c(dG) standard). The total number of 8-oxo-dG = 663 × 10^6^ × 8-oxo-dG/dG.

The analysis of m^5^C was performed as previously described [52]. Briefly, 1 μg of dried DNA was dissolved in a succinate buffer (pH 6.0) containing 10 mM CaCl_2_, and digested with 0.001 units of spleen phosphodiesterase II and 0.02 units of micrococcal nuclease. Next, the digested DNA was radiolabeled using the reaction mixture of 1 μCi [γ-^32^P]ATP (6000 Ci/mM; Hartmann Analytic GmbH), 1.5 units of T4 polynucleotide kinase in 3 μL of 10 mM bicine-NaOH pH 9.7 buffer containing 10 mM MgCl_2_, 10 mM DTT, and 1 mM spermidine. After 30 min at 37 °C, 3 μL of apyrase (10 units/mL) was added to the same buffer and incubated for another 30 min. Next, the 3’ nucleotide phosphate (from [γ-^32^P]dNp) was cleaved off with 0.2 μg RNase P1 in 500 mM ammonium acetate buffer, pH 4.5. The identification of [γ-^32^P]dN was performed by two-dimensional thin-layer chromatography on cellulose plates (Merck; #105577, Burlington, MA, USA) using solvent system: isobutyric acid:NH_4_OH:H_2_O (66:1:17 v/v) in the first dimension, and 0.1 M sodium phosphate (pH 6.8)-ammonium sulfate-n-propyl alcohol (100 mL/60 g/1.5 mL) in the second dimension. Radioactive spot analysis was done with the Phosphoimager Typhoon Screen (Pharmacia, Sweden) and ImageQuant software (GE Healthcare, Chicago, IL, USA). For precise calculation, we used the amount of material in spots corresponding to m^5^dC (5-methylcytosine), dC (cytosine), and dT (thymine). The total m^5^C contents were calculated as R = (m^5^dC/(m^5^dC+dC+dT)) × 100. 

### 4.3. SA-β-gal and Crystal Violet Staining

A histochemical staining kit (Sigma; CS0030-1KT) was used in accordance with the manufacturer’s protocol to detect SA-β-gal activity. Briefly, cells were grown in a T25 flask, rinsed once with PBS, and fixed with 2% formaldehyde and 0.2% glutaraldehyde for 8 min. Following another wash with PBS, cells were incubated overnight at 37 °C in the β-galactosidase staining solution. SA-β-gal+ cells were counted manually and the mean ± SD were calculated from 3 different fields of view, from images containing ≥200 cells. For crystal violet staining, cells were grown in round culture dishes and fixed in 100% methanol. The fixed cells were then incubated for 2 h, in a staining solution that consisted of 47.5 mL of 70% ethanol combined with 2.5mL of 1% aqueous crystal violet solution. After decanting the staining solution, the cells were washed several times in excess water and left to dry at room temperature.

### 4.4. Western Blotting

Cells were washed in PBS (Sigma; 806552), followed by their direct lysis on the culture dish using RIPA buffer (Sigma; R0278), containing protease and phosphatase inhibitors (Roche; #05892970001, #04906845001, Basel, Switzerland). Protein concentrations were determined by bicinchoninic acid protein assay (Sigma; BCA1-1KT), and then adjusted to equal concentrations using lysis buffer, followed by the addition of an SDS loading buffer that contained 2-mercaptoethanol. Lysates (15–20 μg) were electrophoresed in Mini-PROTEAN TGX Stain-Free Gels (Bio-rad; Cat.#4568083, Hercules, CA, USA) in Tris/Glycine/SDS Buffer (Bio-rad; Cat. #1610732). Gel contents were transferred to polyvinylidene difluoride (PVDF) membranes (Bio-rad; Cat.#1620174, Merck Millipore; Cat No. IPVH07850), using a Bio-rad Trans-Blot Semi-dry Transfer Cell. Membranes were blocked in 5% non-fat milk (Bio-rad; Cat. #170-6404), followed by the addition of primary and secondary antibodies. The following commercially available antibodies were used at the indicated concentrations for Western blot; Jmjd3 (Genetex; Cat No. GTX31466 and GTX124222 (1:200)), Utx (Genetex; Cat No. GTX121246 (1:200)), H3K27me3 (Genetex; Cat No. GTX121184 (1:200)), Histone H3 (Sigma; H016 (1:200)), lamin B1 (Invitrogen; Cat.#33-2000 (1:200), Carlsbad, CA, USA), β-actin (Sigma; A1978 (1:1000)), anti-rabbit IgG (whole molecule)–peroxidase antibody produced in goat (Sigma; A6154 (1:6000), Anti-mouse IgG (whole molecule)–peroxidase antibody produced in rabbit (Sigma; A9044 (1:6000)). Membranes were incubated in SuperSignal West Pico PLUS Chemiluminescent Substrate (Thermo Scientific #34580), and imaged and analyzed using the Biorad ChemiDoc XRS+ imaging system. Densitometry was calculated using BioRad Image Lab 6.0 software.

### 4.5. shRNA Knockdown and GSK-J4 Inhibitor Treatment

The TRIPZ inducible lentiviral shRNA plasmids, non-silencing shRNA control, Jmjd3-shRNA, Utx-shRNA (Dharmacon; RHS4743, 301324, 387205), along with the packaging plasmids pCMV-dR8.2 dvpr and pCMV-VSV-G (Addgene; 8455, 8454), were purified using a QIAGEN Plasmid Maxi Kit (Qiagen; Cat No. 12163). Lentiviral particles were produced in 293TN cells (obtained from System Biosciences #LV900A-1) grown in DMEM high glucose with L-glutamine. Briefly, 293TN cells growing in a T75 flask were transfected with the combination of 8 µg of shRNA construct, 4 µg of pCMV-dR8.2 dvpr, and 2 µg of pCMV-VSV-G plasmid, using Lipofectamine 2000 reagent (Invitrogen; #11668) in accordance with the manufacturer’s protocol. Then, 48 h post-transfection, the conditioned media were harvested and filtered using a 0.45-micron PES filter (Millipore; Cat. No. SLHP033RS). The lentiviral particles were stored at −80 °C freezer until use. The transduction of growing MRC5 cells was carried out for 6 h in a T25 flask by adding 1 mL of lentiviral particles to an existing 3 mL of culture media, along with hexadimethrine bromide (Sigma; H9268), at a final concentration of 4 µg/mL. After cell transduction, fresh media was added to allow the cells to recover for 24 h. Stable cell lines were selected for over 10 days by adding a concentration of 1.8 µg/mL puromycin (Sigma-Aldrich; P8833) to the culture media. Then, shRNA expression was induced using 2 µg/mL doxycycline (Sigma; D1822), which was replenished every 24 h by adding directly to the media. GSK-J4 inhibitor (Sigma-Aldrich; SML0701) was resuspended in DMSO (Sigma-Aldrich; 276855), at a stock concentration of 50 mM. For inhibitor treatment experiments, GSK-J4 was replenished every 24 h, by adding it directly to the existing culture media.

### 4.6. RNA Isolation, cDNA Synthesis, and qRT-PCR

RNA was isolated using TRI Reagent (Sigma Aldrich) according to the manufacturer’s protocol. A total of 2 µg of RNA was used for cDNA synthesis using a cDNA Synthesis Kit (iScript Reverse Transcription Supermix for RT-qPCR, Bio-Rad). The reaction conditions for cDNA synthesis were as follows: 5 min at RT, 30 min at 46 °C, 1 min at 95 °C, hold at 4 °C, stored at −20 °C. Quantitative real-time PCR (qRT-PCR) was performed on a Bio-Rad CFX96 cycler using Bio-Rad SYBR Green Mix, in accordance with the manufacturer’s protocol. Reaction conditions for qRT-PCR were as follows: 3 min at 95 °C, followed by 39 cycles of 15 s at 95 °C, 30 s at 68 °C, and 20 s at 72 °C. Then, ΔCt values were calculated using 18S rRNA and HPRT as references. The following oligonucleotides were used:

JMJD3: 5’TGCTCCGTCAACATCAACATTGGC and 5’TGCACGAAGCGGTACACAGGAATA;
UTX: 5’GAACAGCTCCGCGCAAATAG and 5’CGTACCTGTGCAACTCCTGT;
IL6: 5’CCAGGAGAAGATTCCAAAGATGTA and 5’CGTCGAGGATGTACCGAATTT;
IL8: 5’CACAACCCTCTGCACCCAGTTT and 5’GAGAGTGATTGAGAGTGGACCAC;
HPRT: 5’CTTGAGCACACAGAGGGCTACA and 5’CATTATGCTGAGGATTTGGAAAGG;
18S: 5’CGGCTACCACATCCAAGGAA and 5’GCTGGAATTACCGCGGCT.

### 4.7. DNA and Immunofluorescence Staining

To detect the presence of nuclear blebbing and SAHF, cells were fixed for 15 min in 100% methanol pre-chilled at −20 °C, washed in PBS, and incubated with Hoechst 33342 and NucBlue reagent (Molecular Probes; Cat. No. R37605, Eugene, OR, USA) for 1 h at room temperature. To detect F-actin stress fibers, cells were stained with Phalloidin-Atto 488 (Sigma-Aldrich; 49409). For immunofluorescence, cells were fixed in 100% methanol pre-chilled at −20 °C, and then permeabilized for 10 min in a PBS solution containing 0.25% Triton X-100. The cells were then incubated in a 5% BSA blocking solution for 1 h. After blocking, primary antibodies, anti-HA (Sigma H6908), anti-FLAG (Sigma F7425), anti-c-Myc (Sigma; A3956), were added to a 1% BSA solution and incubated with cells overnight at 4 °C. Cells were then incubated with secondary antibody, anti-Rabbit IgG—Atto 488 (Sigma; 18772) for 2 h at room temperature. All wash steps were performed with PBS. Fluoroshield mounting media (Sigma F6182) and a coverslip were added to prepare and preserve some of the samples. All immunostained cells were imaged by fluorescence microscopy.

### 4.8. DNA Constructs and Transfection

The following DNA constructs were obtained from Addgene. First, pCMVHA EED wt (#24231), pCMVHA hEZH2 (#24230), pCMV-HA-JMJD3 (#24167), pCMV-HA-UTX (#24168) were gifts from Kristian Helin. Moreover, pcDNA3/Myc-DNMT1 (#36939), pcDNA3/Myc-DNMT3A (#35521), pcDNA3/Myc-DNMT3L (#35523) were gifts from Arthur Riggs. FH-TET1-pEF (#49792), and FH-TET3-pEF (#49446) were gifts from Anjana Rao. The isolation of the plasmid DNA from bacteria cultures was performed using Gene Elute Plasmid Miniprep Kit (Sigma; PLN70-1KT). For transfections, 293T cells were plated in a 12-well cell culture plate. Once the cells reached 80% confluency, they were transfected with 0.5 μg of DNA using Lipofectamine 3000 (Invitrogen; L3000015), according to the manufacturer’s instructions. The following day, the medium was aspirated from the plate and replaced with 250 μL/well of fresh supplemented EMEM. After 48 h, the cells were subjected to further analysis.

### 4.9. RNA-Sequencing and Analysis

RNA was isolated from MRC5 cells using TRI Reagent (Sigma; T9424). Samples were treated with DNAse I and RNA quality was characterized by an Agilent 2100 Bioanalyzer. Library preparation and sequencing were performed using the NextSeq 550 system (Illumina; SY-415-1002, San Diego, CA, USA). The differential gene expression analysis was calculated using the ‘DESeq2’ package [53]. Up- and down-regulated genes were analyzed using gene ontology (GO) [54]. 

## 5. Conclusions

Senescence reprogramming can have context-dependent tumor-suppressive and oncogenic functions. This study identifies oxidative stress and senescence reprogramming in cancer stem cell formation. We made several observations about the effect of SCM, a chemically defined media, on fibroblasts and cancer cells. We conclude that Jmjd3 and Utx function to control growth arrest and cell fate during SCM-induced senescence, and the aberrant epigenetic regulation of H3K27me3 is associated with the cancer stem cell phenotype.

## Supplementary Materials

The following are available online at https://www.mdpi.com/2072-6694/12/7/1745/s1, Table S1: RNA-seq analysis at each time point. Excel spreadsheet containing the lists of all genes in the datasets for D1, D3, and D5 time points, Table S2: RNA-seq timecourse analysis. Excel spreadsheet containing the list of all genes up- and down-regulated. The timecourse analysis is relative to D0 and combines the datasets for D1, D3, and D5, Table S3: Gene Ontology supplementary data. Excel spreadsheet containing detailed information for the GO pathways identified and the curated lists of up and down-regulated genes with ≤ log2-fold change value of 2, and *p*-value ≤ 0.05 used for the analysis.
